# New Insights for Oxidative Stress and Diabetes Mellitus

**DOI:** 10.1155/2015/875961

**Published:** 2015-05-12

**Authors:** Kenneth Maiese

**Affiliations:** Laboratory of Cellular and Molecular Signaling, Newark, NJ 07101, USA

## Abstract

The release of reactive oxygen species (ROS) and the generation of oxidative stress are considered critical factors for the pathogenesis of diabetes mellitus (DM), a disorder that is growing in prevalence and results in significant economic loss. New therapeutic directions that address the detrimental effects of oxidative stress may be especially warranted to develop effective care for the millions of individuals that currently suffer from DM. The mechanistic target of rapamycin (mTOR), silent mating type information regulation 2 homolog 1 (*S. cerevisiae*) (SIRT1), and Wnt1 inducible signaling pathway protein 1 (WISP1) are especially justified to be considered treatment targets for DM since these pathways can address the complex relationship between stem cells, trophic factors, impaired glucose tolerance, programmed cell death pathways of apoptosis and autophagy, tissue remodeling, cellular energy homeostasis, and vascular biology that greatly impact the biology and disease progression of DM. The translation and development of these pathways into viable therapies will require detailed understanding of their proliferative nature to maximize clinical efficacy and limit adverse effects that have the potential to lead to unintended consequences.

## 1. Oxidative Stress, Cellular Survival, and Programmed Cell Death

Oxidative stress can significantly negatively impact cellular survival and longevity and lead to programmed cell death [1–4]. The generations of reactive oxygen species (ROS) that result in oxidative stress include nitrogen based free radical species such as nitric oxide and peroxynitrite as well as superoxide free radicals, hydrogen peroxide, and singlet oxygen [5]. ROS can result in DNA damage, mitochondrial and other organelle injury, protein misfolding, and neuronal synaptic dysfunction [6–10]. Protective pathways serve to alleviate damage from ROS and involve vitamins B, C, D, and K [11–15], coenzyme Q10 [16], glutathione peroxidase [15, 17], and superoxide dismutase [8, 9, 18–26].

Oxidative stress can lead to the induction of programmed cell death through apoptosis and autophagy [27–31]. Apoptosis has an early phase with the loss of plasma membrane lipid phosphatidylserine (PS) asymmetry and a later phase that leads to genomic DNA degradation [32–34]. Blockade of the early phase with membrane PS externalization is vital for cellular survival since membrane PS externalization can direct inflammatory cells to engulf and remove injured cells [35–37] that may be functional and available for repair [8]. The later phase of cell death with apoptosis leads to the destruction of cellular DNA [1, 38–42].

Autophagy is another pathway of programmed cell death that permits cells to recycle cytoplasmic components while removing dysfunctional organelles for tissue remodeling [30, 34, 43–45]. Of the three categories for autophagy, microautophagy employs the invagination of lysosomal membranes for the sequestration and digestion of cytoplasmic components [34]. In chaperone-mediated autophagy, cytosolic chaperones transport cytoplasmic components across lysosomal membranes. The most prevalent category of autophagy is macroautophagy that consists of the sequestration of cytoplasmic proteins and organelles into autophagosomes. These autophagosomes then combine with lysosomes for degradation and are subsequently recycled for future cellular processes [27, 46–49].

## 2. Diabetes Mellitus and Clinical Implications

Diabetes Mellitus (DM) is affecting a greater proportion of the world's population each year such that the World Health Organization predicts that DM will be the seventh leading cause of death by the year 2030 [50]. As of the year 2013, almost 350 million individuals are estimated to suffer from DM. In the United States, twenty-one million individuals have DM [51] and an additional eight million individuals are estimated to be presently undiagnosed with DM [52, 53]. The costs of caring for individuals with DM are significant. The United States in the year 2012 spent $8,915 per person for healthcare and approximately seventeen percent of the country's Gross Domestic Product per the Centers for Medicare and Medicaid Services (CMS) [54]. In relation to DM, $176 billion was spent for direct medical costs and another $69 billion in lost finances resulted from reduced productivity.

Early diagnosis and proper care of individuals with DM also may be crucial for extending human longevity by modulating epigenetic changes in age-related genes involved with DM and other degenerative disorders [55–59]. The presence of impaired glucose tolerance in the young raises additional concerns for the future development of DM in these individuals [60]. Obesity is another risk factor for the development of DM [29, 60–63]. Obesity results in cellular oxidative stress and insulin resistance [64, 65], altered trophic factor release [66–69], lipid-induced impairment of pancreatic *β*-cells [42], and dysfunctional protein tyrosine phosphatase signaling [63, 70].

In insulin dependent (Type 2) DM, defective insulin secretion can result from impaired *β*-cell function, oxidative stress [29, 71], the absence of inhibitory feedback through plasma glucagon levels, chronic exposure to free fatty acids [72], lipotoxicity [42], and hyperglycemia [8]. Type 2 DM is the most prevalent subtype for this disorder occurring in ninety percent of individuals that are usually over the age of 40. A progressive deterioration of glucose tolerance occurs with early *β*-cell compensation that is followed by a decrease in pancreatic *β*-cell mass with insulin resistance and impaired insulin secretion [29].

In contrast to Type 2 DM, Type 1 DM is an autoimmune disorder with the presence of alleles of the human leukocyte antigen (HLA) class II genes within the major histocompatibility complex (MHC). Type 1 DM occurs in approximately 5–10% of patients with DM [29]. Activation of T-cell clones that are capable of recognizing and destroying pancreatic *β*-cells to result in insulin deficiency may not always lead to programmed cell death but rather relies upon the necrotic death of *β*-cells [73]. Destruction of pancreatic *β*-cells with inflammatory infiltration of the islets of Langerhans results in the loss of insulin production and regulation. Almost all patients with Type 1 DM have increased titers of autoantibodies (Type 1A DM). However, approximately 10% of Type 1 DM individuals do not have serum autoantibodies and are considered to have maturity-onset diabetes of the young (MODY) that can be a result of *β*-cell dysfunction with autosomal-dominant inheritance (Type 1B DM). Type 1 and Type 2 DM may have common links since approximately 10% of individuals with Type 2 DM may have elevated serum autoantibodies similar to Type 1 DM [74] and insulin resistance also may be a component of Type 1 DM in some patients [75–77].

DM essentially affects all systems of the body. In the vascular system, high glucose leads to the loss of endothelial cells [69, 71, 78–82], fosters endothelial cell senescence [83], prevents angiogenesis [84], impairs mobilization of bone marrow endothelial progenitor cells [85], injures the neuroglialvascular unit injury [86], and results in diffuse vascular disease [27, 81, 83, 84, 87–89]. DM results in additional vascular events that result in cardiac disease [46, 90–97], atherosclerosis [2], and renal disorders [61, 98–101]. DM leads to immune system dysfunction [12, 96, 102–105], liver disorders [106–109], stroke [7, 14, 59, 87, 110, 111], Alzheimer's disease [68, 75, 112, 113], psychiatric disease [114, 115], visual loss [86, 116–118], and peripheral nerve impairment [88].

## 3. Diabetes Mellitus, Oxidative Stress, and Programmed Cell Death

Progressive disease in the body that occurs during DM is mediated to a significant extent through the release of ROS and oxidative stress [8, 14, 29, 119–123]. Patients with Type 2 DM have serum markers of oxidative stress with ischemia-modified albumin [124]. Acute rises in serum glucose as well as chronic elevations can result in the release of ROS during DM [125]. In addition, some studies suggest that treatment with antioxidants may limit the prevention of cardiovascular disease during DM [14].

In cell culture models of DM, elevated glucose levels result in oxidative stress and cell injury in cardiomyocytes [93, 97, 126], endothelial cells [78–80, 85, 86, 127], and neurons [21, 71, 116, 128, 129]. Oxidative stress also results in elevated glutathione levels and increased lipid peroxidation in murine animal models of Type 2 DM [92]. Advanced glycation end products (AGEs), entities that foster complications in DM [91], lead to the release of ROS and caspase activation [122].

At the cellular level, uncoupling proteins (UCPs), a family of carrier proteins found in the inner membrane of mitochondria and consist of the mammalian members UCP1, UCP2, UCP3, UCP4, and UCP5, can significantly influence cell survival in DM [76, 120, 130]. UCPs uncouple oxygen consumption through the respiratory chain from ATP synthesis [48, 76, 120, 131]. Subsequently, this leads to oxidative stress as UCPs disperse a proton electrochemical potential gradient across the mitochondrial inner membrane resulting in the activation of substrate oxidation and dissipation of oxidation energy as heat instead of ATP [76, 120]. Uncoupling of respiration by UCPs modulates ATP synthesis, fatty acid release, and glucose oxidation. Overexpression of UCP in skeletal muscle of mice enhances responsiveness to insulin, improves glucose transport in skeletal muscle, and increases resistance to obesity [132]. In addition, skeletal muscle respiratory uncoupling can improve insulin sensitivity in obesity [133]. In regards to UCP3, it can stimulate insulin uptake [134], can facilitate fatty acid oxidation, and can limit ROS production [135]. However, it should be recognized that not all UCPs are beneficial. Overexpression of UCP2 in isolated pancreatic islets leads to decreased ATP levels and reduced glucose-stimulated insulin secretion. Loss of UCP2 improves insulin secretion and decreases hyperglycemia in leptin-deficient mice [136].

Closely tied to the role of UCPs is the impairment of mitochondrial dysfunction that can occur during oxidative stress [2, 5, 6, 137, 138]. Skeletal muscle mitochondria in patients with Type 2 DM have been reported to be smaller than those in control subjects [139]. Decreased mitochondrial proteins and mitochondrial DNA in adipocytes also have been associated with the development of Type 2 DM [140]. Exposure of glucolipotoxicity to pancreatic *β*-cells promotes oxidative stress and mitochondrial dysfunction with cytochrome c release, caspase activation, and apoptosis [141]. Mitochondrial dysfunction leads to the opening of the mitochondrial membrane permeability transition pore, release of cytochrome c, and subsequent caspase activation [27, 78, 80, 82, 127, 141, 142].

The pathways of programmed cell death that involve apoptosis [14, 82, 88, 143, 144] and autophagy [8, 34, 61, 145] also regulate cell survival during DM and oxidative stress. For example, “highly oxidized glycated” low density lipoproteins that are formed during DM result in oxidative stress in human retinal capillary pericytes with subsequent induction of apoptosis and autophagy [116]. Current studies also suggest that the programmed cell death pathway of necroptosis may not significantly contribute to cell injury during DM, but future work may change this perspective [146]. In murine models of Type 1 DM, necroptosis may have less than an essential role in cell survival during DM [73]. In relation to apoptosis, apoptotic cell death during DM occurs in pancreatic *β*-cells [147–149], cardiomyocytes [46, 93, 96], endothelial cells [79, 83, 84, 127, 150], renal cells [151–153], and neurons [21, 114, 128, 154].

Autophagy may be cytoprotective as well as detrimental to cell survival during DM. During elevated glucose, autophagy can impair endothelial progenitor cells, lead to mitochondrial oxidative and endoplasmic reticulum stress [155], and prevent the formation of new blood vessels [27]. Increased activity of autophagy has been associated with loss of cardiac and liver tissue in diabetic rats during attempts to achieve glycemic control through diet modification [156]. AGEs also have been shown to lead to the induction of autophagy and vascular smooth muscle proliferation that can cause atherosclerosis [89] as well as cardiomyopathy [126]. Yet, under some conditions, autophagy may be less of a significant mediator of cell injury [157] and it may actually be beneficial. Autophagy may be required to eliminate misfolded proteins and nonfunctioning mitochondria to avert *β*-cell dysfunction and the onset of DM [141]. Loss of autophagy with haploinsufficiency of an essential* Atg7* gene in murine models of obesity can lead to increased insulin resistance with elevated lipids and inflammation [47]. Autophagy also may improve insulin sensitivity during high fat diets in mice [65] and autophagy has been associated with exercise in mice that regulates glucose homeostasis [158]. Pathways of autophagy and apoptosis also can work in unison to modulate cell survival. Induction of autophagy may protect cardiomyocytes from apoptotic cell death during DM [46].

## 4. Mechanistic Target of Rapamycin

**Figure 1:**
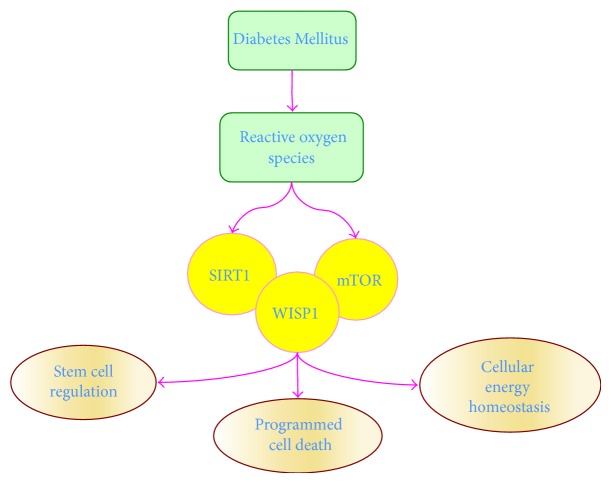
Oxidative stress in Diabetes Mellitus impacts pathways of stem cell proliferation, programmed cell death, and cellular energy homeostasis. Diabetes Mellitus (DM) leads to the development of oxidative stress and the release of reactive oxygen species (ROS). Novel proliferative pathways for targeting new treatments against DM and the complications of this disorder are the mechanistic target of rapamycin (mTOR), silent mating type information regulation 2 homolog 1 (*S. cerevisiae*) (SIRT1), and Wnt1 inducible signaling pathway protein 1 (WISP1). Each of these pathways is intimately connected through shared signal transduction mechanisms that can oversee stem cell proliferation, programmed cell death that involves apoptosis and autophagy, and cellular energy homeostasis that can affect mitochondrial function and insulin sensitivity.

The mechanistic target of rapamycin (mTOR), also termed the mammalian target of rapamycin and FK506-binding protein 12-rapamycin complex-associated protein 1, is a principal pathway in DM that can significantly affect apoptosis and autophagy [2, 91, 159, 160] (Figure 1). mTOR is a 289-kDa serine/threonine protein kinase. It is encoded by a single gene* FRAP1* [161–163] and is a component of the protein complexes mTOR Complex 1 (mTORC1) and mTOR Complex 2 (mTORC2) [164, 165]. mTORC1 consists of Raptor (Regulatory-Associated Protein of mTOR), the proline rich Akt substrate 40 kDa (PRAS40), Deptor (DEP domain-containing mTOR interacting protein), and mammalian lethal with Sec13 protein 8 (mLST8). mTORC2 consists of Rictor (Rapamycin-Insensitive Companion of mTOR), Deptor, mLST8, the mammalian stress-activated protein kinase interacting protein (mSIN1), and the protein observed with Rictor-1 (Protor-1) [165, 166].

mTOR is a vital component of cytokine and growth factor signaling such as erythropoietin (EPO) [66, 167–171] (Table 1). EPO uses mTOR for cytoprotection [61, 91, 100, 165, 172]. Through mTOR, EPO protects vascular cells [67, 173], prevents cell injury during *β*-amyloid (A*β*) exposure [170], modulates bone homeostasis [167], promotes retinal progenitor cell survival during oxidant stress [169] and blocks retinal degeneration in models of polycystic kidney disease [86], promotes the neuronal phenotype of adult neuronal precursor cells [168], improves cognitive function in sepsis-associated encephalopathy [171], and limits cell injury during oxygen-glucose deprivation [174, 175]. In regards to cellular metabolism, EPO promotes wound healing during DM [102], maintains cellular mitochondrial function and energy metabolism [82], reduces the detrimental effects of obesity in animal models [69], limits high glucose-induced oxidative stress in renal tubular cells [151], and protects endothelial cells during experimental models of DM [78, 79].

Cytokines and growth factors are not the only agents that rely upon the regulation of mTOR in DM to impact cellular survival. Metformin, an agent that controls hyperglycemia in DM, inhibits mTOR activity and promotes autophagy. Metformin oversees mTOR activity through AMP activated protein kinase (AMPK). AMPK controls the activity of the hamartin (tuberous sclerosis 1)/tuberin (tuberous sclerosis 2) (TSC1/TSC2) complex that is an inhibitor of mTORC1 [176]. AMPK phosphorylates TSC2 as well as Raptor to block the activity of mTOR and the complex mTORC1 during energy stress [177]. By activating AMPK to inhibit mTOR, metformin prevents cell loss during hypoxia [178], reduces cardiomyopathy in experimental models of DM [179], increases cardiomyocyte cell survival [46], and protects cortical brain tissue during cerebral ischemia [110]. Through mTOR inhibition, metformin prevents endothelial cell senescence [83], blocks neuronal apoptotic cell death [180], and prevents androgen upregulation during prostate cancer [181]. AMPK activity can be protective against hypoxia [178], promote autophagy to reduce memory impairment [182], and reduce oxidative stress that can lead to vascular hypertension [183]. Yet, AMPK activity is not consistently beneficial and can cause aberrant A*β* stress [184] and A*β* toxicity [185], cardiac tissue hypertrophy [186], and neuroinflammation [187].

Independently, mTOR activation controls stem cell development [2, 16, 165, 176, 188–190], promotes pancreatic *β*-cell proliferation [149], prevents apoptosis, limits insulin resistance, blocks vascular thrombosis in patients with metabolic syndrome [191], and prevents pathways associated with atherosclerosis [192] (Table 1). In addition, mTOR activation through application of glucagon-like peptide-1 agonists can protect pancreatic *β*- cells from cholesterol mediated apoptotic cell injury [72]. mTOR also functions through the epidermal growth factor receptor to prevent neuronal apoptotic cell loss during DM [154].

## 5. Silent Mating Type Information Regulation 2 Homolog 1 (*S. cerevisiae*)

Silent mating type information regulation 2 homolog 1 (*S. cerevisiae*) (SIRT1), also known as NAD-dependent deacetylase sirtuin-1, is a member of the Sirtuin family and plays a significant role in cellular metabolism and DM (Figure 1). As histone deacetylases, sirtuins transfer acetyl groups from *ε*-N-acetyl lysine amino acids on the histones of DNA to regulate cellular transcription [193–195]. SIRT1 can modulate cellular metabolism [105, 196–198] and may be critical for the development of new therapies for DM [29, 52, 193, 199]. SIRT1 is one of seven mammalian homologues of the yeast silent information regulator-2 (Sir2) that control posttranslational changes of proteins for cellular growth and maintenance [83, 127, 199, 200]. SIRT1 is dependent upon NAD^+^ as a substrate [105, 193, 201, 202] and through nicotinamide phosphoribosyltransferase (NAMPT) catalyzes the conversion of nicotinamide to nicotinamide mononucleotide [12]. Nicotinamide mononucleotide is subsequently converted to NAD^+^ by the nicotinamide/nicotinic acid mononucleotide adenylyltransferase (NMNAT) enzyme family [203]. NMNAT can control the deacetylating activity of SIRT1. NAMPT activity increases cellular NAD levels and enhances the activity of SIRT1 transcription. Mammalian forkhead transcription factors can also influence SIRT1 activity [193, 204–206]. Mammalian forkhead transcription factors bind to the SIRT1 promoter region, a cluster of five putative core binding repeat motifs (IRS-1) and a forkhead-like consensus-binding site (FKHD-L) [207]. This binding fosters the ability of forkhead transcription factors, such as FoxO1, to control SIRT1 transcription and increase SIRT1 expression [208].

SIRT1 importantly modulates stem cell survival that may ultimately influence cellular protection during DM as well as other cellular toxic environments [7, 48, 209]. Recent work suggests that stem cell strategies may be effective for maintaining glucose homeostasis during DM in animal models [210, 211]. SIRT1 is required for telomere elongation and genomic stability of induced pluripotent stem cells [212] (Table 1). During oxidative stress, SIRT1 prevents apoptosis through the induction of autophagy in endothelial progenitor cells [213]. SIRT1 can regulate autophagic flux [214] to promote the transition of muscle stem cells from a quiescence state to an active state [215]. Mesenchymal stem cells with SIRT1 overexpression lead to increased blood vessel density in the area of cardiac infarcts, reduced cardiac remodeling, and improved cardiac performance in rodent models [216]. Increased SIRT1 expression also enhances the survival of cardiomyoblasts [217]. These studies of cardiovascular protection suggest an important role for angiogenesis especially since patients with Type 2 DM show a downregulation of endothelial progenitor cells that has been associated with decreased SIRT1 protein levels [218]. In particular, SIRT1 prevents senescence and impaired differentiation in endothelial progenitor cells [219] and is necessary for the angiogenic properties of human mesenchymal stem cells [220]. SIRT1 also is necessary for endothelial progenitor cell mobilization and vascular repair during DM in mice [196]. SIRT1 can preserve angiogenesis in rodent models of DM with bone marrow-derived early outgrowth cells [221]. In addition, SIRT1 may function in conjunction with growth factors to foster improved cardiac performance during glucose depletion through the activation of aged mesenchymal stem cells [222].

In some cases, a limited activity of SIRT1 may be required for optimal stem cell function. SIRT1 can be a negative regulator of subventricular zone and hippocampal neural precursors in murine animal models. Knockdown of SIRT1 does not eliminate neural precursor numbers but increases the production of neurons in the subventricular zone and the hippocampus [223]. Absence of SIRT1 with the induction of heat shock protein-70 (HSP70) also is necessary to promote neural differentiation, maturation of embryonic cortical neurons [224], and the differentiation of human embryonic stem cells into motoneurons [225]. Neuronal differentiation also can be driven through the microRNA miR-34a that leads to decreased SIRT1 expression and DNA-binding of p53. However, a minimum level of SIRT1 may be necessary for some cells since increased expression of SIRT1 can increase the astrocytic subpopulation of cells that are necessary to support neuronal cell populations [226].

In mature and differentiated cells, SIRT1 can prevent insulin resistance through a number of mechanisms that involve fat mobilization [197], mTOR signaling [227], and control of cellular inflammation [228] (Table 1). SIRT1 can increase insulin signaling in insulin-sensitive organs through pathways that involve phosphoinositide 3-kinase (PI 3-K) and protein kinase B (Akt) [32, 127, 200, 229–231]. SIRT1 also can stimulate glucose-dependent insulin secretion from pancreatic *β*-cells by repressing UCP2 [232]. Loss of SIRT1 can lead to insulin resistance and excessive hepatic lipid accumulation [197]. Gene deletion or pharmacological inhibition of SIRT1 can impair insulin signaling by interfering with insulin stimulated insulin receptor phosphorylation and glycogen synthase [233]. Overexpression of SIRT1 has been shown to decrease hepatic steatosis and improve insulin sensitivity [108]. Interestingly, SIRT1 has been shown to increase lifespan in higher organisms such as* Drosophila* and offer protection against oxidative stress [234], and it is used by EPO to prevent cell injury during oxidative stress and DM. EPO can increase endogenous cellular SIRT1 activity and promote the subcellular nuclear trafficking of SIRT1 to result in endothelial cell protection during oxidative stress [200]. EPO is able to maintain adipose cell energy homeostasis and protect against metabolic disorders such as DM through SIRT1 [198].

SIRT1 promotes cellular survival during oxidative stress and DM by preventing the induction of apoptotic pathways. Loss of SIRT1 activity in human mesenchymal stem cells leads to reduced cellular proliferation with increased apoptosis [220]. Absence of SIRT1 in mouse cochlear neurons and in the auditory cortex also is associated with hearing loss [235]. Decreased levels of SIRT1 that occurs in smokers and chronic obstructive disease patients lead to endothelial progenitor cell dysfunction with apoptotic cell death [236]. In contrast, SIRT1 activation can prevent neuronal apoptosis in models of traumatic brain injury [237]. SIRT1 activation can protect endothelial progenitor cells against apoptosis [238] and enhance skeletal myoblast survival [239] during tumor necrosis factor-*α* (TNF-*α*) exposure. SIRT1 can prevent the externalization of membrane PS residues during apoptosis [32, 127, 200, 240]. As a result of the cytoprotective capacity of SIRT1, mechanisms that can block SIRT1 degradation are vital. Pathways involving Wnt signaling have been shown to prevent SIRT1 degradation and block caspase activation [241–244]. Decreased levels of SIRT1 activity can be the result of apoptotic pathways associated with p38 [245] and c-Jun N-terminal kinase-1 (JNK1) [106]. Caspase degradation of SIRT1 [246] also can lead to further activation of caspases [246, 247]. As described, loss of SIRT1 activity can yield significant consequences for cellular protection. Absent or insufficient SIRT1 activity can be detrimental for vascular cells [127, 200, 248], prevent protection against cardiovascular disease [249], and lead to neuronal injury [231, 243, 250]. Yet, in some cases, a reduction in SIRT1 activity may be necessary to promote cellular survival such as in studies involving trophic factors with insulin growth factor-1 [251].

Autophagy also is another significant component in determining cell survival with SIRT1. SIRT1 promotes autophagy in mitochondria [252] that may be required to maintain a healthy mitochondrial pool associated with cellular metabolism [253]. SIRT1 activation is able to limit apoptotic cell injury and improve cognition through the induction of autophagy in models of cognitive loss that employ chronic intermittent hypoxia hypercapnia exposure [254]. SIRT1 leads to the induction of autophagy for chondrocyte survival during oxidative stress. Loss of autophagy through knockdown of the forkhead transcription factors FoxO1 and FoxO3 depletes SIRT1 activity and results in chondrocyte cell death [18]. However, in some scenarios, SIRT1 protection has been reported to involve the downregulation of autophagy. SIRT1 blocks cell injury through the inhibition of autophagy in pulmonary models of oxidative stress during exposure to cigarette smoke in bronchial epithelial cells [255, 256].

Intimately tied to the ability of SIRT1 to modulate autophagy are the pathways of AMPK and mTOR (Table 1). AMPK increases NAMPT during glucose restriction leading to increased NAD^+^ [257] and decreased levels of the SIRT1 inhibitor nicotinamide [60]. Resveratrol, an activator of SIRT1, can enhance AMPK activity through SIRT1 dependent and independent mechanisms [258, 259]. Once active, AMPK can phosphorylate TSC2 and inhibit mTORC1 activity [8, 165]. Through AMPK activity, SIRT1 inhibits mTOR. As a result, SIRT1 promotes autophagy to protect embryonic stem cells during oxidative stress [190]. SIRT1 has been shown to inhibit mTOR signaling to promote neuronal growth [260] and assist with mesangial cell proliferation during high glucose exposure [261]. AMPK also can increase the cellular NAD^+^/NADH ratio leading to the deacetylation of downstream SIRT1 targets that include the peroxisome proliferator-activated receptor-gamma coactivator 1 (PGC-1*α*), FoxO1 [94], and FoxO3a [258]. SIRT1 upregulation with AMPK activation promotes autophagy that is necessary for endothelial cell protection during exposure to oxidized low density lipoproteins that can lead to atherosclerosis [206]. SIRT1 also uses AMPK for the regulation of insulin sensitivity. Endothelial cell protection from oxidized low density lipoproteins requires SIRT1 as well as AMPK activation [206, 262]. SIRT1 activation with AMPK also may be necessary to protect against spatial memory impairment in combined experimental models of DM and Alzheimer's disease. Loss of SIRT1 and AMPK activities can lead to cognitive loss, oxidative stress, and neuronal cell apoptosis [112].

## 6. Wnt1 Inducible Signaling Pathway Protein 1

The CCN family member Wnt1 inducible signaling pathway protein 1 (WISP1) has cellular signaling pathways linked to mTOR and SIRT1 that highlight the significance of WISP1 in DM (Figure 1). The CCN family of proteins consists of six secreted extracellular matrix associated proteins and are defined by the first three members of the family that include cysteine-rich protein 61, connective tissue growth factor, and Nephroblastoma overexpressed gene [263]. Members of this family such as WISP1 contain four cysteine-rich modular domains that include insulin-like growth factor-binding domain, thrombospondin domain, von Willebrand factor type C module, and C-terminal cysteine knot-like domain [264]. The* WISP1* gene was identified in a mouse mammary epithelial cell line [265] and subsequently demonstrated to modulate gastric tumor growth [266]. WISP1 is a matricellular protein [267] and a downstream target of the* wingless* pathway Wnt1 that has broad cellular effects to control programmed cell death, stem cell growth, immunity, nervous, cardiovascular, and musculoskeletal system development, and tumorigenesis [79, 268–280]. WISP1 can control cell survival through pathways that involve autophagy [34, 157], apoptosis [243, 281–283], and caspase activation [243, 282, 284]. During oxidative stress, WISP1 can upregulate PI 3-K and Akt [157, 243, 284] and protect against A*β* exposure [185], cardiomyocyte injury [282], and DNA damage [281] (Table 1). Through Akt, WISP1 also leads to fibroblast proliferation in airway remodeling [285], vascular smooth muscle proliferation [286], inhibitory phosphorylation of glycogen synthase kinase-3*β* (GSK-3*β*) [157, 282, 284, 285], and the maintenance of *β*-catenin that can prevent apoptotic cell death [36, 269, 274, 287, 288].

Similar to pathways involving mTOR and SIRT1, a portion of the protective capacity of WISP1 may rely upon stem cell oversight. WISP1 can influence induced pluripotent stem cell reprogramming [289]. WISP1 is differentially regulated during stem cell migration and stem cell differentiation. WISP1 expression is increased during stem cell migration [290] and repressed in adipose-derived stem cells during hepatic differentiation [291]. WISP1 expression that may be associated with inflammation and obesity is increased during human adipocyte differentiation [292]. During pancreatic regeneration, WISP1 is one of several genes that are overexpressed, suggesting that WISP1 may be reparative during DM [293]. WISP1 also may be vital for therapeutic strategies against vascular disease in DM. WISP1 leads to vascular smooth muscle proliferation that may be important for tissue repair as well as affect restenosis following vascular grafting [286, 294]. WISP1 may provide support for vascular repair and regeneration during saphenous vein crush injury [295]. WISP1 also oversees cellular senescence [296] and does not lead to excessive cellular proliferation in aging vascular cells [297] that can lead to atherosclerosis (Table 1).

In regards to mTOR and WISP1 [264, 275], WISP1 activates and phosphorylates mTOR. WISP1 also leads to the activation of the mTOR signaling pathways of p70 ribosomal S6 kinase (p70S6K) and the eukaryotic initiation factor 4E-binding protein 1 (4EBP1) [91, 174]. WISP1 increases mTOR activity by antagonizing the inhibitory actions of the mTOR component proline rich Akt substrate 40 kDa (PRAS40) [298]. WISP1 also oversees the posttranslational phosphorylation of AMPK that is involved in glucose homeostasis [164, 165, 299, 300]. WISP1 controls AMPK activation by differentially decreasing phosphorylation of TSC2 at Ser^1387^, a target of AMPK, and increasing phosphorylation of TSC2 at Thr^1462^, a target of Akt [185]. The ability of WISP1 to modulate AMPK activity is vital for the regulation of cellular metabolism during DM [300]. AMPK activity can reduce insulin resistance and lessen oxidative stress through activation of autophagy [65]. AMPK also may prevent myocardial ischemia in experimental models of DM [301], promote proper metabolic function of cells [302], and place limits on adipocyte differentiation, lipid accumulation, and obesity [262]. Yet, the level of AMPK activity is a significant consideration in DM. In some experimental models of Type 2 DM, AMPK activation can lead to apoptosis in pancreatic islet cells [303].

WISP1 also regulates cellular metabolism through its protective actions over SIRT1. WISP1 increases SIRT1 activity and fosters SIRT1 nuclear translocation [243] to block apoptotic cell injury [127, 200, 304]. WISP1 controls the mammalian forkhead transcription factor FoxO3a that is involved in cellular metabolism to block caspase activity [42, 94, 107, 305, 306] and prevent the degradation of SIRT1 during oxidative stress [243] (Table 1). As previously described, Wnt signaling that involves WISP1 is vital to prevent SIRT1 degradation, prevent caspase activation, and promote cellular survival [241–244].

## 7. Conclusions

Oxidative stress is a significant mediator of multisystem disease in the body during DM. Clinical studies and experimental models point to cell injury that involves both apoptosis and autophagy during DM as a result of oxidative stress and the release of ROS. Given that DM is predicted to become the seventh leading cause of death by the year 2030, the need for new therapeutic opportunities to treat DM and its complications becomes increasingly acute. One exciting strategy for consideration is mTOR. mTOR activation oversees stem cell development, fosters pancreatic *β*-cell proliferation, limits insulin resistance, and can prevent pathways that may lead to atherosclerosis. Protective cytokines and growth factors such as EPO rely upon mTOR for vascular cell protection, neuronal cell survival, and bone homeostasis. Furthermore, EPO can lead to wound healing during DM, maintains cellular mitochondrial function and energy metabolism, and reduces the detrimental effects of obesity in animal models. Yet, inhibitory pathways of mTOR that involve AMPK also have a critical role during DM. AMPK activity can reduce insulin resistance and lessen oxidative stress through activation of autophagy. In addition, metformin, an agent that controls hyperglycemia in DM, activates AMPK and inhibits mTOR activity to promote autophagy and cytoprotection. Metformin reduces cardiomyopathy in experimental models of DM, prevents endothelial cell senescence, and prevents neuronal apoptotic cell death. Interestingly, SIRT1 also relies upon AMPK for the regulation of insulin sensitivity and to induce autophagy that is necessary for endothelial cell protection during exposure to oxidized low density lipoproteins that can lead to atherosclerosis. Yet, AMPK activity is not consistently beneficial and can lead to A*β* stress, A*β* toxicity, cardiac tissue hypertrophy, and neuroinflammation. In some experimental models of Type 2 DM, AMPK activation can lead to apoptosis in pancreatic islet cells. SIRT1 importantly modulates stem cell survival, blocks apoptotic cell injury, controls autophagy for mitochondrial pool maintenance, and limits oxidative stress that affects cellular survival during DM. Although SIRT1 can increase cell survival and preserve insulin signaling by blocking apoptotic pathways, SIRT1 also can foster autophagy and limit mTOR activation to preserve mitochondria, promote stem cell proliferation, and prevent insulin resistance. WISP1 incorporates the pathways of mTOR and SIRT1 to control stem cell migration as well as stem cell differentiation. WISP1 may offer protection against cell loss in DM since it is one of several transcripts that are expressed during pancreatic regeneration. WISP1 can activate PI 3-K, Akt, and mTOR to protect against A*β* exposure, cardiomyocyte injury, DNA damage, and oxidative stress. WISP1 also increases SIRT1 activity and maintains the integrity of SIRT1 during oxidative stress to prevent SIRT1 degradation. New insights that develop mTOR, SIRT1, and WISP1 as effective therapeutic strategies against DM offer great hope for the millions of individuals that presently suffer from this disabling disorder. Fruits of such investigations will weigh heavily upon careful analysis of the intricate and complex pathways controlled by the proliferative properties of mTOR, SIRT1, and WISP1 to achieve high clinical efficacy for patients with DM and limit adverse effects that can involve organ dysfunction, pancreatic cell loss, tumor growth, and inflammation.

## Figures and Tables

**Table 1:** Targeting mTOR, SIRT1, and WISP1 in Oxidative Stress and DM.

Target	Actions
mTOR	Functions through trophic factors such as EPO to protect vascular cells, promotes neuronal precursors, and blocks retinal degeneration Functions through metformin during mTOR inhibition and AMPK activity to protect against cardiomyopathy, protect cortical brain tissue, and prevent endothelial senescence Promotes stem cell development, promotes pancreatic *β*-cell proliferation, and blocks vascular thrombosis

SIRT1	Promotes telomere elongation and genomic stability of induced pluripotent stem cells, prevents apoptosis in endothelial progenitor cells and mesenchymal stem cells, increases lifespan in higher organisms, and preserves angiogenesis Prevents insulin resistance through fat mobilization, mTOR signaling, and control of cellular inflammation Increases insulin signaling through PI 3-K and Akt and insulin release in pancreatic cells, promotes autophagy to protect embryonic stem cells during oxidative stress, in conjunction with AMPK activation protects endothelial cells during exposure to oxidized low density lipoproteins that can lead to atherosclerosis

WISP1	During pancreatic regeneration, WISP1 is one of several genes that are over-expressed, suggesting that WISP1 may be reparative during DM Promotes vascular smooth muscle proliferation that may be important for tissue repair, activates PI 3-K and Akt pathways to protect cells against oxidative stress and programmed cell death Oversees vascular senescence, modulates AMPK activity that may be sometimes detrimental, maintains the integrity of SIRT1 and prevent its degradation during oxidative stress

AMPK: AMP activated protein kinase; Akt: protein kinase B; EPO: erythropoietin; mTOR: mechanistic target of rapamycin; PI 3-K: phosphoinositide 3 –kinase; SIRT1: silent mating type information regulation 2 homolog 1 (*S. cerevisiae*); WISP1: wnt1 inducible signaling pathway protein 1.
